# A standardised faecal collection protocol for intestinal helminth egg counts in Asian elephants, *Elephas maximus*

**DOI:** 10.1016/j.ijppaw.2015.06.001

**Published:** 2015-06-30

**Authors:** Carly L. Lynsdale, Diogo J. Franco dos Santos, Adam D. Hayward, Khyne U. Mar, Win Htut, Htoo Htoo Aung, Aung Thura Soe, Virpi Lummaa

**Affiliations:** a Department of Animal and Plant Sciences, Alfred Denny Building, University of Sheffield, Western Bank, Sheffield S10 2TN, UK; b Department of Animal Health, Faculty of Veterinary Medicine, University of Lisbon, Avenida da Universidade Técnica, 1300-477 Lisbon, Portugal; c Institute of Evolutionary Biology, University of Edinburgh, The King's Buildings, Ashworth Laboratories, Charlotte Auerbach Road, Edinburgh EH9 3FL, UK; d Myanma Timber Enterprise, Ministry of Environmental Conservation and Forestry, Gyogone Forest Compound, Bayint Naung Road, Insein Township, Yangon, Myanmar

**Keywords:** Faecal egg count, Parasite, Sampling method

## Abstract

The quantitative assessment of parasite infection is necessary to measure, manage and reduce infection risk in both wild and captive animal populations. Traditional faecal flotation methods which aim to quantify parasite burden, such as the McMaster egg counting technique, are widely used in veterinary medicine, agricultural management and wildlife parasitology. Although many modifications to the McMaster method exist, few account for systematic variation in parasite egg output which may lead to inaccurate estimations of infection intensity through faecal egg counts (FEC). To adapt the McMaster method for use in sampling Asian elephants (*Elephas maximus*), we tested a number of possible sources of error regarding faecal sampling, focussing on helminth eggs and using a population of over 120 semi-captive elephants distributed across northern Myanmar. These included time of day of defecation, effects of storage in 10% formalin and 10% formol saline and variation in egg distribution between and within faecal boluses. We found no significant difference in the distribution of helminth eggs within faecal matter or for different defecation times, however, storage in formol saline and formalin significantly decreased egg recovery. This is the first study to analyse several collection and storage aspects of a widely-used traditional parasitology method for helminth parasites of *E. maximus* using known host individuals. We suggest that for the modified McMaster technique, a minimum of one fresh sample per elephant collected from any freshly produced bolus in the total faecal matter and at any point within a 7.5 h time period (7.30am–2.55 pm) will consistently represent parasite load. This study defines a protocol which may be used to test pre-analytic factors and effectively determine infection load in species which produce large quantities of vegetative faeces, such as non-ruminant megaherbivores.

## Highlights

• We tested effects of sampling method on FECs of helminth burdens in *Elephas maximus*.
• Storage in fixative solution significantly reduced FECs.
• Sample origin and time of sample collection had no effect on FECs.
• We outline a standardised sampling protocol for *E. maximus*.
• We encourage testing potential pre-analytical sources of error in other host taxa.

## Graphical abstract

**Figure:**
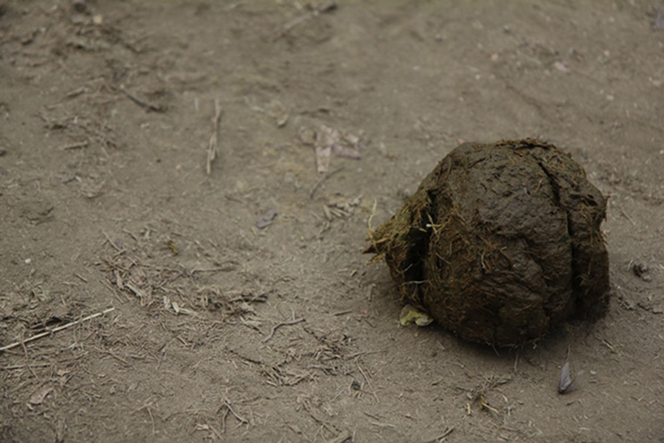

## 1 Introduction

Helminth parasites are found ubiquitously across vertebrate taxa and pose substantial threats to the welfare, management and conservation of both natural and captive populations (Pedersen et al., 2007; Zhang et al., 2008). This is especially true for endangered or endemic species, with infection often interacting with additional factors, such as poaching or habitat fragmentation, to drive population decline (Cleaveland et al., 2009; Heard et al., 2013). Coprological techniques, such as faecal flotation, form the basis of gastrointestinal helminth egg detection and estimation of parasite abundance within individual hosts. Faecal flotation can allow for microscopic identification and quantification of helminth eggs in a faecal sample, which is typically expressed as a faecal egg count (FEC) quantified in eggs per gram of faeces (EPG) (MAFF, 1986). FEC is only an estimate of parasite burden, subject to between-host variation in parasite development and population structure, as well as adult parasite sex ratio, number and fecundity (Guyatt and Bundy, 1993; Tompkins and Hudson, 1999). On a host level, host sex, age and location may influence infection and cause variation in FEC (Döpfer et al., 2004; Wood et al., 2013; Lyons et al., 2014). Nevertheless, it is an essential tool for quantifying gastro-intestinal helminth burden where invasive methods (such as post-mortem sampling or endoscopy) are impractical, and has been proven to provide a reliable estimate of individual parasite burdens in a range of host species (Roberts and Swan, 1981; Seivwright et al., 2004; Denwood et al., 2012). Estimation of helminth burdens is also key for designing appropriate treatment or management regimes in captive host populations (Coles et al., 1992; Döpfer et al., 2004) and for better understanding patterns of infectious disease and current health status of wild populations (Jolles et al., 2008).

Whilst molecular approaches such as real-time PCR are becoming increasingly popular in the field of quantitative parasitology (Zarlenga and Higgins, 2001), they are difficult to apply outside of laboratory conditions. For parasitologists in remote field locations or without routine access to specialist molecular tools, traditional parasitology methods such as faecal flotation are more tractable. It is therefore vital to improve the accuracy and reliability of current quantitative protocols in order to reduce potential sources of error where possible as well as investing in alternative modern techniques. While a variety of flotation approaches are available, such as FLOTAC (Cringoli, 2006, 2010) and Cornell–Wisconsin (Egwand and Slocombe, 1982), the McMaster technique (MAFF, 1986) is inexpensive and easily replicable, and remains one of the most frequently employed methods for use in wildlife parasitology (Coles et al., 1992; Gillespie, 2006; Hing et al., 2013; Thomas and Morgan, 2013; Stringer et al., 2014). Various published modifications take into account differences in available equipment, required sensitivity of diagnosis, range of available flotation solutions and different coefficients for interpretation (MAFF, 1986; Roepstorff and Nansen, 1998; Cringoli et al., 2004; Morrison, 2004; Elsheikha and Khan, 2011; Vadlejch et al., 2011). More recently, publications have focused on certain pre-analytical factors which may affect method reliability (Vidya and Sukumar, 2002; Nielsen et al., 2010; Stringer et al., 2014). Many previous studies have detected the presence of infection and quantified nematode burden using the McMaster technique. However, the majority of such studies which focus on wild host taxa have done so without establishing the distribution of eggs through the host-specific faecal matter or investigating pre-analytic effects, such as storage of faecal samples, on egg recovery.

There is ambiguity in the literature as to whether nematode egg distribution is constant within faecal matter, or within an individual host (MAFF, 1986; Yu et al., 1998; Vidya and Sukumar, 2002; Abeysinghe et al., 2012; Denwood et al., 2012). The range of species which act as hosts to helminth parasites is highly diverse and methodological error may be increased through using a generalised faecal sampling protocol without accounting for potentially substantial differences in host faeces size, composition and consistency. For larger vertebrates, it is impractical to account for non-uniform egg output through adjusting sample size: for example, adult Asian elephants (*Elephas maximus*) may produce over 18 kg of faeces in one defecation event. Variation in egg distribution could be controlled for by homogenising faeces or by combining samples using a composite-like method as in Nicholls and Obendorf (1994) and Morgan et al. (2005), but only for single defecations. However, this is difficult with non-ruminant herbivores such as elephants, rhinoceros and equine species due to the non-viscous, vegetative structure of faecal matter produced.

A further source of bias in FEC may be introduced by the fact that faecal samples may be collected from free-ranging hosts opportunistically across the day, as it is uncertain whether variability may exist in shedding rates of parasites over a 24 h period (MAFF, 1986; Warnick, 1992; Carstensen et al., 2012). In such instances time of sample collection may be a potential source of error. Finally, when data collection is carried out in field sites or under extreme weather conditions, storage of samples in fixative solutions is used until analysis can be completed. However certain sample storage methods have been found to significantly distort the FEC for some internal parasites (Moitinho et al., 1999). In summary, accurate quantification of FEC may depend upon (1) the distribution of eggs within faecal matter; (2) time of sample collection; and (3) storage method of faecal samples.

In this study, we aim to determine controllable pre-analytical factors which may affect FEC in Asian elephants in order to design a species-specific protocol for reliably estimating within-host helminth burdens. Relatively few studies have investigated parasitic infection within Asian elephants, with a variety of approaches implemented in the absence of a standardised sampling protocol (Watve and Sukumar, 1997; Vidya and Sukumar, 2002; Vanitha et al., 2011; Abeysinghe et al., 2012; Hing et al., 2013). Of the gastro-intestinal helminths, nematodes are one of the most commonly-recorded elephant parasites and heavy infections can cause damage to the host. These effects can lead to increased risk of secondary infections, diarrhoea, reduced digestion efficiency, malnutrition and emaciation, stunted growth and anaemia (Fowler and Mikota, 2006). Myanmar is home to the second-largest wild population of Asian elephants and the largest remaining captive population world-wide (Sukumar, 2006), with over 2700 captive, government owned individuals currently engaged in the logging industry (Mar, 2007). Of all recorded deaths in the Myanmar timber elephant population, 21% are linked to infection (Mar, 2007; Mar et al., 2012; Mumby et al., 2013a) with parasite-induced pathology likely to contribute to existing high mortality rates (Mumby et al., 2013b) and low fertility rates (Mumby et al., 2013; Lahdenperä et al., 2014) in the timber elephants. Consequently, the working elephant population is not currently self-sustaining, with the annual capture of wild conspecifics necessary to supplement the timber elephant workforce and meet workloads (Mar, 2007).

It is therefore paramount that hosts such as *E. maximus* are sampled in a consistent manner that allows helminths FEC to be obtained as accurately as possible in order to quantify infection burden and design appropriate control strategies. A species-specific protocol for quantifying helminth FEC, which minimizes pre-analytical sources of error, must be established before the health and fitness impact of these parasites on their hosts can be reliably determined. In this study we develop such a protocol in a large sample of known individually marked, semi-captive Asian elephants working in Myanmar. We assess the distribution of gastro-intestinal helminth eggs within faecal matter by quantifying FEC in samples from (1) different parts of a single bolus and (2) between multiple boluses. We determined effects of time of collection by (3) quantifying FEC in samples collected at two different time points during a 7.5 h period. Finally, we assessed (4) impact of storage in two common fixatives (10% formol saline and 10% formalin) by comparing FEC in samples collected from the same location within a single faecal bolus, with one sample being analysed as fresh without being stored in fixative and the other after being stored in preservative before analysis. Our study provides Asian elephant-specific adaptations of the special modification of the McMaster method, with standardised collection and storage guidelines in order to improve reliability of this long-standing method of assessing individual parasite burden.

## 2 Materials and methods

### 2.1 Study population

This study utilizes a semi-captive population of working timber elephants located in two areas of Sagaing region, northern Myanmar; Katha (24°10′ N, 96°19′ E) and Kawlin (23°46′ N, 95°40′ E). Myanmar is home to approximately 10,000 Asian elephants (thought to be at least 20% of the current global total), with the government-owned working population comprising over 20% of the national estimate and over half of Myanmar's total captive population (Sukumar 2006, IUCN, 2014). Most of the working elephants in Myanmar are government-owned and are each assigned a four digit personal identification number, which is permanently marked on their haunches, as well as an individual log book detailing life-history, health and reproductive data from birth to death (Mar, 2002, 2007). This allows for accurate identification of each individual with consistent monitoring of maternal lineages as well as known health and life-history events (Robinson et al., 2012; Hayward et al., 2014). The employment of the elephants is contracted from June–February, with March–May being a period of rest during the hottest months of the year. The elephants work during the day and at night they are free to roam and forage in the surrounding forest, where they also interact and mate with both wild and captive conspecifics. In this study, we collected a total of 820 samples from 129 individual elephants (72 females and 57 males, aged 4–63 years) between November 2013–June 2014.

### 2.2 General sample collection

Defecation was observed for individual elephants, which were easily recognisable by their unique identification numbers. A whole faecal bolus (the last produced within a dung pile unless otherwise stated) was collected and dissected immediately upon observing defecation. Boluses were only taken from the ground and not from any standing water bodies. Fresh faecal samples were collected manually by splitting boluses in half and obtaining a large volume of faecal matter from central and edge locations of one half. From these, smaller subsamples weighing exactly 4.5 g were collected. Fresh samples were kept in sealed and labelled zip-locked bags and analysed as soon as possible after collection. If fresh samples could not be analysed immediately after collection they were stored in a cooler box for a maximum of 8 h and transferred to a fridge kept at 4–6 °C. Fridge samples were analysed within 7 days, in accordance with Nielsen et al. (2010).

### 2.3 Egg counting technique

Adapting the special modification of the McMaster method designed specifically for helminthology (as outlined in MAFF, 1986), samples of 4.5 g of faeces were weighed and mixed thoroughly in 40.5 ml of saturated salt (NaCl) solution (which has an approximate specific gravity of 1.20, Cringoli et al., 2004). This was then strained using a sieve with an aperture width of approximately 1 mm and the debris discarded. Then, 0.5 ml of the resultant solute was transferred into a double-chambered McMaster slide, mixing the solute again before pipetting into the second chamber. The slide was then left for 5 min to allow all faecal debris with a specific gravity higher than 1.20 to sink and helminth eggs (specifically nematodes which can be recovered using a saturated salt flotation solution, Cringoli et al., 2004; MAFF, 1986; Taylor et al., 2007) to float to within a visible microscopic range.

The chambers were examined microscopically using a compound microscope under 10× magnification. All eggs observed within the two separate chambers (both inside and outside the marked grid) were counted to obtain a faecal egg count (FEC). A measure of eggs per gram (EPG) was calculated by multiplying FEC by 10, providing a measure with a resolution of 10 EPG. We arrived at a multiplication factor of 10 by dividing the faeces:NaCl solution dilution factor (1 in 10, therefore a dilution factor of 10) by the total volume of solute examined (two chambers at 0.5 ml each, with a total volume of 1 ml). Helminth eggs were visually identified to phylum level through recognition of descriptive characteristics as stated or depicted in MAFF (1986) and Taylor et al. (2007).

### 2.4 Statistical analysis

All statistical analyses were carried out using R version 3.1.1 (R Development Core Team, 2014). All Generalised linear mixed effects models (GLMMs) were run using the lme 4 package (version 1.1–7, Bates et al., 2014). Diagnostic plots of model residuals were investigated to establish the goodness of fit for all models, which showed an appropriate fit of the data for the relevant error structure used in each instance.

### 2.5 Parasite egg distribution within faecal matter

Asian elephants can produce a large quantity of faecal matter during a single defecation, normally in the form of 5–8 distinct faecal boluses (Cheeran, 2002). To determine if nematode eggs were evenly distributed within faecal matter produced during a defecation, differences in FEC of faeces were assessed by carrying out faecal egg counts from 1) samples collected from different locations within a single bolus (at a ‘within-bolus’ scale) and 2) samples collected from different faecal boluses (at a ‘between-bolus’ scale).

To determine egg distribution within a single bolus, four faecal samples were collected from two separate locations (two from the centre and two from nearest the outside edge) for 117 individuals (with three faecal samples instead of four collected for two elephants, for whom only one centre sample was collected). These individuals were spread across the two study sites with samples collected between November–December 2013. Two faecal samples were collected per location (‘centre’ and ‘edge’) from the last faecal bolus produced during a single defecation. Care was taken not to collect any of the exterior surface when collecting edge samples, which were located at least 0.5 cm below the surface, to avoid contamination of samples from ground-dwelling helminths. Gloves were worn during sample collection and changed or cleaned after each collection event to prevent cross-contamination between samples.

To investigate differences in egg distribution between multiple boluses, samples were collected from the first, middle and last bolus expelled during a single defecation. Two samples (one centre and one edge) were collected from each bolus from a total of 20 individual elephants across the two study sites in March–April 2014.

To statistically investigate the relationship between FEC and sample origin we implemented GLMMs accounting for Poisson-lognormal distribution (with the function glmer). We first constructed GLMMs with FEC (raw count) as the response variable, with fixed effects of elephant sex (binary), age group (factor with 4 levels; calves at heel 0–4 years; trained calves >4–16 years; working adults >16–53 years; retired adults >53 years) and study site (as a two-level factor). A random effect of individual identification number (elephant ID) was included to control for individual variation and repeated measurements in the same individual. An additional random effect assigning an individual level to each data point (an observation-level random effect) was included in all models. This consisted of consecutive numerical values describing row number of the raw data. This accounted for the overdispersion within the response variable (faecal egg count) in accordance with Poisson-lognormal model structure (Elston et al., 2000; Harrison, 2014).

Faecal samples originated either from different locations (centre and edge) within a single bolus (at a ‘within-bolus’ scale) or from different faecal boluses (first, middle or last bolus, at a ‘between-bolus’ scale). To establish whether sample origin (at both within and between bolus scales) had a significant effect on FEC, we compared the base models with (1) models including a two-level fixed factor for within bolus location (centre or edge) and (2) a three-level fixed factor for between bolus location (first, middle or last bolus). Between-bolus models also included a separate two-level fixed factor for location, as used for within bolus models (centre or edge). Models were compared using likelihood ratio tests (LRTs) where the χ^2^ test statistic is calculated as −2*(LogLik_model1_ − LogLik_model2_), with the p value calculated on either 1 (for within bolus) or 2 (for between bolus) degrees of freedom. This tested for sample origin effects accounting for all of these sources of variation. We then returned to the original model and tested the significance of sex, age, site and both random factors using LRTs, retaining only significant terms. Following removal of non-significant terms (see Supplementary files), the effect of sample origin was removed from the reduced model and again tested using an LRT. The between bolus model was then re-levelled so that each level of the sample origin fixed factor (first, middle or last bolus) was each used as the model reference category, with the other levels tested against it. Here and elsewhere, we did not have an *a priori* biological reason to predict the effect of our main term of interest (here within or between bolus location of the sample) to differ between the sexes, ages, or study locations, and such interactions were therefore not included in any models.

### 2.6 Effect of time of sample collection

In order to establish if faecal egg counts from samples collected at different times of day were dissimilar, two defecation events were sampled for each elephant between a 7.5 h period (earliest approximately 7.30 am local time and latest 2.55 pm local time), the first collected in the morning (‘AM’) and one collected after midday (‘PM’). Time of defecation was recorded, with one centre and one edge sample collected at each defecation event (AM and PM) for 47 individual elephants across both study sites during March 2014 and June 2014.

GLMMs with Poisson-lognormal errors, again with raw faecal egg count as a response variable, were used to establish differences in FEC of samples collected at different times of day (from different defecations). A single measure of FEC was used per time point in the analysis, with FEC calculated as the mean of the centre and edge samples from within a single bolus. As before models accounted for elephant ID, sex, age and study site as categorical variables. Age was included as a continuous variable (as opposed to as a factor, as above) to aid model convergence. We tested whether the effect of age was linear or non-linear by comparing models where FEC followed a linear or quadratic trajectory with age; age as quadratic term was added to the starting model structure, with a reduced model compared to the original using an LRT as previously described. Individual elephant identification number and an observation level effect (as previously described in Section 2.5) were included as random factors. We then added a two-level categorical fixed effect comparing collection times: the first FEC was from a sample collected before 12 pm local time (‘AM’ sample) and the second collected after 12 pm local time (‘PM’ sample). We tested for an association with time of collection by comparing models with and without the effect of collection time in the model, using LRTs as above. Finally, we then returned to the original model and tested the significance of age (included as both linear and quadratic terms), sex, study site and random factors using LRTs, removing the non-significant terms. We then compared this reduced model with a comparable model which excluded the effect of time of sampling, again using an LRT, in order to determine the effect of sampling time accounting only for other significant fixed effects.

### 2.7 Effect of storage in fixative

To investigate the impact of storage of samples in fixative solution on faecal egg counts, 33 elephants were sampled from both study sites during December 2013 and March 2014. A larger amount of faecal matter was collected for centre and edge samples and split into two measured subsamples of 4.5 g each, one being analysed directly (as fresh) and the other stored in either 10% formol saline or 10% formalin (to a minimum ratio of 1:3 parts faeces:fixative). Stored samples were left for a period of at least 5 days and kept out of direct sunlight or artificial light. Upon analysis the samples were thoroughly mixed, sieved and then centrifuged at 1500 rpm for 5 min. After centrifugation, the solute was discarded and the remaining pellet of faecal matter fully resuspended in saturated salt (NaCl) solution with any large clusters of matter broken by manual homogenisation and pipetting. The faecal suspension was then pipetted into the double chambered McMaster slide and eggs were counted, as described above in Section 2.3.

To statistically establish how FEC was affected by storage we used GLMMs with Poisson-lognormal errors with raw FEC values. We compared samples analysed as fresh versus those kept in either formol saline or formalin for at least 5 days following collection. As before, models accounted for elephant ID, sex, age and study site as categorical variables with age split by working classes as defined in parasite egg distributional analysis. In addition, individual elephant identification number and an observation-level effect were included as random factors. Storage was included as a three-level categorical fixed factor, with categories classified as 1) fresh 2) stored in formalin or 3) stored in formol saline. We compared models including and excluding storage as a fixed factor with an LRT. As above, we then returned to the original model and tested the significance of age, sex, study site and random factors using LRTs, retaining only significant terms. We then tested the effect of storage by comparing the reduced model with a comparable model excluding the storage variable, again using an LRT. We tested the variability of any change in faecal egg counts following storage using two separate GLMMs. We used the function glmer with Poisson-lognormal errors with sample (fresh v. storage method): 1) for samples stored in 10% formalin and 2) for samples stored in 10% formol saline. Both models included a random term which defined a random effect of slope (change in FEC between fresh-stored samples) and intercept (elephant ID). We tested for an association of variation in slope (whether any change in FEC was uniform or not) by comparing models with and without the random slope effect using an LRT. A significant effect would indicate that there was non-uniform change in FEC of stored samples across individual elephants.

## 3 Results

**Fig. 1:**
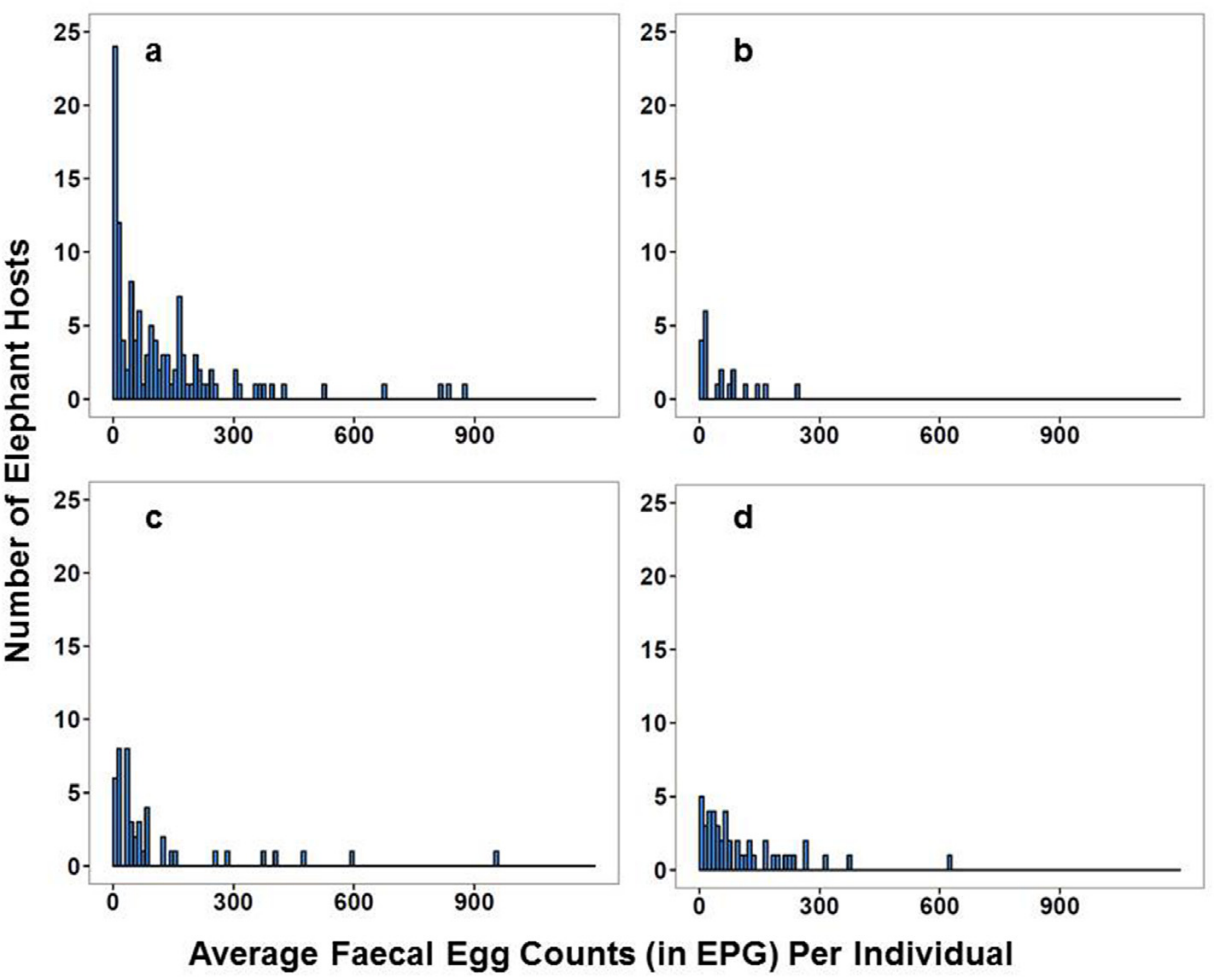
Averaged helminth egg counts for every elephant host sampled for each experiment; investigating egg distribution within (a) an individual bolus (centre and edge samples), 474 samples from 119 elephants and (b) multiple boluses (centre and edge samples from different boluses), 120 samples from 20 elephants, (c) when determining optimal sampling time, 94 samples from 47 elephants, and (d) if storage methods had any impact on egg recovery during faecal egg counts (FEC), 132 samples from 33 elephants. Helminth eggs were always aggregated within host elephants, with few hosts having substantial parasite burdens (in excess of 200 EPG) and the majority having none or insubstantial levels of infection.

Strongyle and *Strongyloides*-type nematode eggs were common in faecal samples, with suspected *Paramphistomum* eggs found occasionally. Faecal egg counts were highly aggregated among individuals, with a high degree of between-individual variation in egg counts (Fig. 1, also see Table S1). All the subsequent results are adjusted for differences between individuals, sexes, ages and study locations. These factors may contribute to variation in faecal matter size and structure, as well as to that in parasite abundance, but were not the primary focus in the current paper. The model estimates are stated ± estimated standard error, adjusted for a Poisson-lognormal distribution and using a log link function. Raw means are given with ±standard error.

### 3.1 Parasite egg distribution within faecal matter

**Fig. 2:**
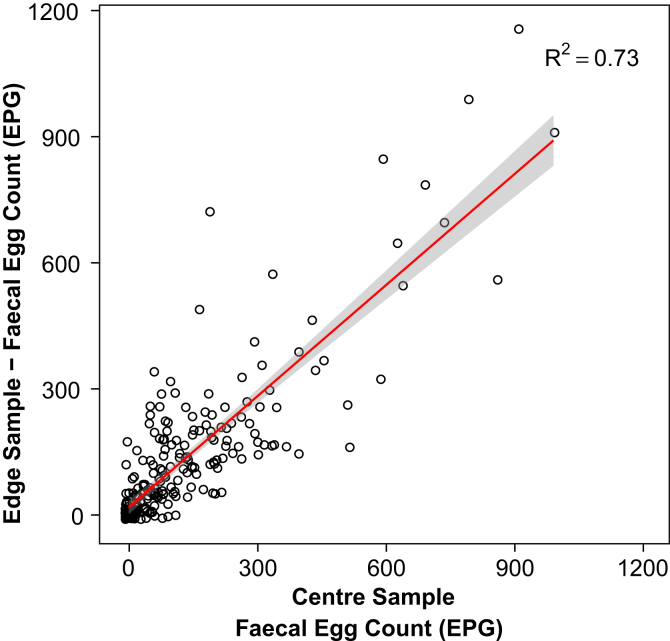
Regression of the faecal egg counts (in EPG) for samples taken of the centre and edge of a single faecal bolus with 95% confidence intervals, 474 samples collected from 119 elephants.

Helminth egg distribution in elephant faeces did not differ according to the locations sampled in a single defecation. This was concordant for samples collected from disparate locations both within a single faecal bolus and those collected from different faecal boluses. There was no significant difference in FEC between samples collected from the centre and edge of the same bolus (centre versus edge estimate = −0.07 ± 0.05; Χ^2^_(1, 474)_ = 1.65, *p* = 0.20) with centre and edge samples being highly correlated (Fig. 2). Accordingly the raw data showed little difference in mean FEC in samples collected from the centre (122.75 ± 10.02 EPG) versus the edge (126.36 ± 10.52 EPG) of the bolus (see Table S1).

**Fig. 3:**
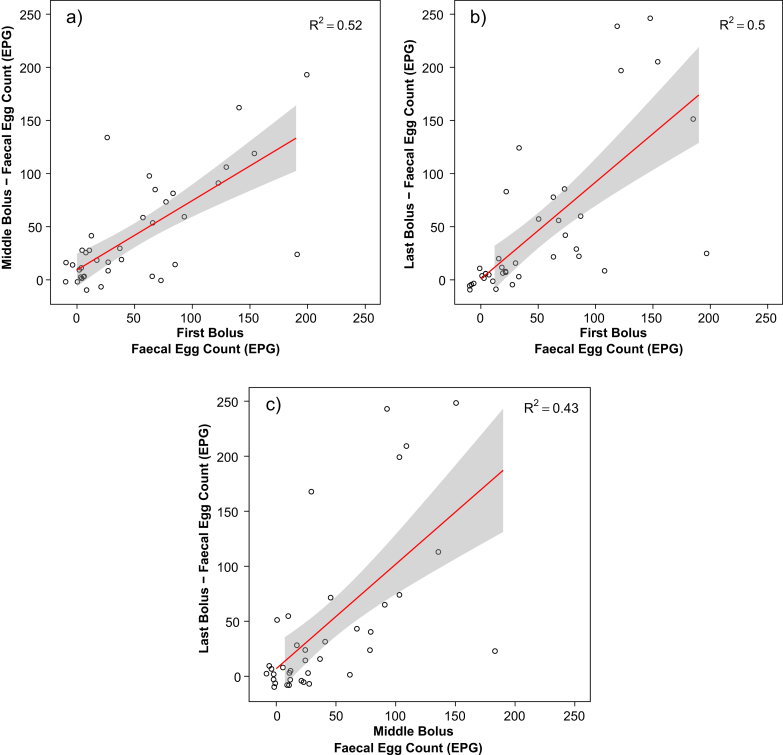
Pairwise comparisons of faecal egg counts (in EPG) for samples taken from different faecal boluses produced in one defecation event of (a) first and middle boluses, (b) first and last boluses, (c) middle and last boluses, all with 95% confidence intervals. For each of (a–c) 40 samples collected from 20 elephants. Data collected for one elephant not shown, with one extreme data point removed in each of a–c, to allow for better presentation of plots.

In addition, there was no significant difference between samples collected from the first (mean = 42.5 ± 9.29 EPG), middle (mean = 54.06 ± 16.65 EPG) or last (mean = 42.19 ± 14.03 EPG) boluses produced during a single defecation (Χ^2^_(2, 120)_ = 0.97, *p* = 0.62), with positive association observed for all of these samples (Fig. 3). These results controlled for significant effects of age category, sex and study site on FECs (Tables S2–S4).

### 3.2 Effect of time of sample collection

The time of sample collection was found to have no significant effect on helminth faecal egg count (PM versus AM estimate = −0.25 ± 0.001, Χ^2^_(1, 94)_ = 1.29, *p* = 0.26, see Table S5). Samples collected in the afternoon had a non-significantly lower raw mean (130.74 EPG ± 36.39) than samples collected in the morning (raw mean = 153.09 ± 61.34).

### 3.3 Effects of storage in fixative solution

**Fig. 4:**
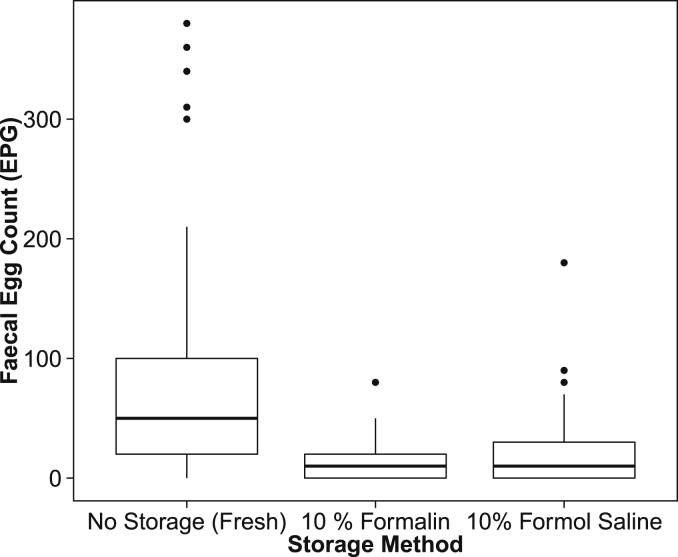
Faecal egg counts (in EPG) were significantly decreased in samples which had been stored in 10% formalin or 10% formol saline in comparison to subsamples collected at the same time but analysed as fresh, without storage in fixative solution. This figure is based on 132 samples collected from 33 elephants, with data lying between the first and third quartiles as represented by the top and bottom horizontal lines of the boxplot. The data range is shown by the vertical black lines, with the median of each dataset represented by the middle horizontal line within each boxplot and with any outliers shown as points.

Storage in fixative solutions was found to have a significant effect on FEC (Χ^2^_(2, 132)_ = 55.90, *p* < 0.001). Nematode egg recovery during faecal egg counts was significantly decreased in samples stored in 10% formalin (estimate = −1.43 ± 0.26) or 10% formol saline (estimate = −1.35 ± 0.19), compared to those analysed as fresh without storage (see Table S6). On average, samples stored in 10% formalin (raw mean = 13.46 ± 3.46 EPG) were found to have an 82.2% decrease in EPG relative to samples which were analysed as fresh without any preservation in fixative solution (raw mean = 75.61 ± 11.34 EPG, Fig. 4). Samples stored in 10% formol saline (raw mean = 23.25 ± 5.53 EPG, Fig. 4) on average showed a 69.25% decrease in FEC when compared to fresh samples. The decrease in FEC following storage in 10% formalin was found to be uniform for all elephants (Χ^2^_(2, 52)_ = 1.02, *p* = 0.60), but not following storage in 10% formol saline (Χ^2^_(2, 80)_ = 11.03, *p* < 0.01).

## 4 Discussion

In this study, we investigated possible sources of bias in a widely used faecal flotation technique in order to develop a standardized protocol for quantifying helminth faecal egg counts in Asian elephants. We investigated three potential sources of error: the distribution of helminth eggs within faecal matter; time of collection; and effect of sample storage in fixative solutions. Our results have enabled us to outline a faecal flotation protocol which considers pre-analytic sources of variation, providing a more reliable estimate of parasite faecal egg count. We found that helminth egg distribution did not differ both within and between the tested faecal boluses, and was independent of time of collection. However, storage in both 10% formalin and 10% formol saline resulted in a marked decrease in FEC. Our findings are meaningful for parasitology studies which necessitate accurate quantitative estimation where invasive or necroscopic assessment is not feasible. This includes those investigating parasitism of endangered host species or those which produce highly voluminous faeces, or faeces with a high vegetative content. Our results will also be of use to future studies considering formaldehyde-based storage methods of faecal samples for use in parasitology.

Our findings are concordant with Vidya and Sukumar (2002) who found parasite egg densities to be uniform within faeces of Asian elephants, but not Stringer et al. (2014) who reported a significantly higher egg count for black rhinoceros (*Diceros bicornis*) in samples collected from the centre of faecal boluses than those collected from the surface. As parasites could be host-specific or limited to specific geographic ranges, this discrepancy in results could be due to different compositions and egg-shedding dynamics of parasite species in the two host species. It could be argued that to test for complete homogeneity within the total faecal matter, every bolus produced within a single defecation should be sampled. However we believe that by obtaining multiple samples from three boluses, from the beginning, midpoint and end of a defecation, our findings are reliably representative of a single defecation event. For the Asian elephants in our population, we conclude that a single faecal sample, collected from anywhere in the faeces, will provide an estimate of parasite abundance which is representative of single defecations of individual elephants. For this semi-captive elephant population, this allows for simpler collection methods henceforth through reduced sampling effort and decreased cost and labour. Our results should be useful in investigations of other host species which produce amounts of faeces similar to or greater than do Asian elephants. These include other non-ruminant, megaherbivorous hosts such as African elephants, *Loxodonta africana* and *Rhinocerotidae* spp., for which non-uniform egg-distribution may be a particular concern.

Faecal samples can be collected at any time of day within the tested 7.5 h window of 7.30am–2.55 pm for the sampled working Asian elephant population, as samples collected at different time points yielded comparable FEC. This is contrary to the findings of Vidya and Sukumar (2002), who suggested that egg densities of Asian elephant hosts differed significantly over 5 h. This could be due to differences in sample size, sample collection and analysis methods or variation in feeding habits of the two sample populations. Looking more broadly across studies on domesticated species, our results are corroborative with those of Carstensen et al. (2012), who observed a lack of significant variation in FEC between faecal samples collected daily from horses (*Equus* spp). In addition Warnick (1992) observed greater than expected fluctuations in daily FEC within horses. However this variation was low enough for samples to still give a reliable indication of population-level infection (when estimating pasture contamination) or for use in prescribing anthelmintic treatment.

The option of storing faecal samples for future analysis is highly desirable when working in challenging field situations, such as in remote locations or during extreme seasons, e.g. monsoons, which may impede the speed and efficiency of sample collection. However, despite numerous parasitology studies utilizing storage of faecal samples in fixative solutions, many have not tested the potential impact of storage on parasite egg recovery, and may be obtaining misleading estimates of parasite abundance. Our findings were congruous with previous studies which have found substantial reductions in FEC following storage in chemical preservatives for parasites of equine and cervid species (Foreyt, 1986; Jagła et al., 2013). Storage heavily impacted upon egg recovery, giving reduced FEC in comparison to those obtained from samples which were not stored in any fixative. It should be noted that although stored samples were centrifuged but fresh samples were not. However, there is support within the literature suggesting that, when correctly executed, centrifugation increases reliability and precision of analysis rather than causing a reduction in FEC (Vadlejch et al., 2011). Therefore, the observed drop in FEC between fresh and stored samples can be attributed to the fixative-induced changes in egg morphology and consequent rupture, which may reduce the floatability and visibility of eggs when observed microscopically in flotation solution. The reduction in faecal egg counts was found to be uniform across all animals for samples stored in 10% formalin. While such samples may be unsuitable for investigating prevalence of infection, they may be used to investigate infection intensity if the consistent drop in FEC is accounted for. However, as the decrease in FEC was not uniform for samples stored in 10% formol saline, samples undergoing this storage treatment are not appropriate for investigating either prevalence or infection intensity. Eggs of helminth species inhabiting Asian elephants and possibly other megaherbivores may therefore be significantly compromised when samples undergo storage in acidic formaldehyde-derived solutions. Studies such as Vanitha et al. (2011) which have implemented such storage methods may have reported inaccurate estimations of FEC for the hosts sampled, if reported storage methods have not been thoroughly tested *a priori*. This effect could be parasite species specific, dependent on egg morphology and structure, with different nematode species affected at differing levels due to, for example, dissimilarities in egg wall thickness. Therefore field protocols, at least sampling Asian elephant hosts, should always utilize fresh samples where possible or investigate the effects of proposed storage on FEC. This conclusion is potentially more widely applicable to other host species, including other non-ruminant megaherbivores, but preliminary research should assess the impact of storage on FEC for different hosts, due to potential variation between parasite species. Furthermore, in the absence of a long-term storage solution, fresh samples can be stored in anaerobic conditions (e.g. in sealed, zip-locked bags) at 4–6 °C for approximately 7 days without significant declines in egg recovery (as outlined in Nielsen et al., 2010). This allows for delayed hatching of helminth eggs but also does not affect egg morphology, floatability and therefore final FEC. It should be noted that error caused through opportunistic sampling of unknown individuals (primarily the potential of mistaking two separate samples produced from the same host as being from two independent hosts) was not a concern as in other studies which sample from unmarked or wild hosts, due to our intimate knowledge of individual animals in this population and direct observation of defecation. Potential temporal, spatial and environmental sources of error were also reduced through sampling host elephants over two different sites, over two different field seasons (each in different seasons with November–December falling in Myanmar's cold season and March–April in the hot season).

Faecal egg counts are the only widely-available tool and remain the sole, practical quantitative method which can be used for non-invasive estimation of gastrointestinal helminth burdens for large, endangered vertebrates. We outline an adapted technique for a population of semi-captive Asian elephants. We found that one faecal sample collected per host, collected from a single bolus at any time between 7:30 am and 2.55 pm was sufficient to give a reliable FEC if analysed when fresh or stored for up to 7 days at 4–6 °C in dark, anaerobic conditions. We urge investigators studying parasitic infections in other host taxa to incorporate similar sources of variation into study design prior to data collection and investigate a variety of sampling methods, as we have done. This will allow for the most effective protocol for each system, accounting for potential methodological sources of error and improving burden estimate accuracy. Our method provides a basis for the experimental design of future studies which may wish to sample extremely far-ranging or exclusive host species, with the outlined protocol having the potential to increase future study application and versatility.
