# Supplementary material for: A standardised faecal collection protocol for intestinal helminth egg counts in Asian elephants, Elephas maximus
Source: Int J Parasitol Parasites Wildl. 2015 Jun 30;4(3):307–15. doi: 10.1016/j.ijppaw.2015.06.001 (PMC4501537; doi:10.1016/j.ijppaw.2015.06.001)
Supplement: Supplementary file 1 [file mmc1.docx]

**Supplementary Files**

**Table S1.** Fixed and random effects estimates from the final generalized linear mixed-effects model with Poisson-lognormal error structure, modelling the effect of faecal sample origin within a single bolus (taken from the centre or edge of the bolus) on helminth faecal egg count (FEC). Only significant terms (tested using likelihood ratio tests, as described in Methods) or terms of interest (sample location) were retained in the final model structure. For the starting model summary table see **Table S7.** X^2^ and associated *p* values are from an LRT comparing models with or without the effect of the tested variable, e.g. sample location (‘Sample’). Estimates are expressed on the log scale. Intercept corresponds to samples originating from the edge of the faecal bolus, for elephants aged 0-4 years (age category 1) and located in Katha. Individual elephant identification number was included as a random effect, as was an observation level random effect (OLRE). The model was fitted to observations from 119 elephants and included a total of 474 samples with a measure of FEC. For all tables, all figures are truncated to 3 decimal places.

| Fixed Effect Coefficients | Estimate | | Std. Error | Χ^2^ | d.f. | Pr(>Chisq) | |
| --- | --- | --- | --- | --- | --- | --- | --- |
| (Intercept) | 0.979 | | 1.711 |  |  |  | |
| Sample (Edge) | 0.000 | | 0.000 | 1.651 | 1 | 0.199 | |
| Sample (Centre) | -0.070 | | 0.054 |  |  |  | |
| Age category 1: <5 years | 0.000 | | 0.000 | 48.090 | 3 | <0.001 | |
| Age category 2: 5-16 years | 1.262 | | 1.702 |  |  |  | |
| Age category 3: 17-53 years | 0.604 | | 1.715 |  |  |  | |
| Age category 4: >53 years | -0.141 | | 1.766 |  |  |  | |
| Camp (Katha) | 0.000 | | 0.000 | 7.765 | 1 | 0.005 | |
| Camp (Kawlin) | -0.876 | | 0.311 |  |  |  | |
| Random Effect Coefficients | Estimate | | St. Dev. | Χ^2^ | d.f. | | Pr(>Chisq) |
| Observation | 0.167 | 0.409 | | 241.430 | 1 | | <0.001 |
| ID | 2.598 | 1.612 | | 481.050 | 1 | | <0.001 |

**Table S2.** All fixed and random effects estimates for a final generalized linear mixed-effects model with Poisson-lognormal error structure modelling the effect of faecal sample origin within different boluses (the first, middle or last bolus produced during a defecation) on helminth faecal egg count (FEC). All original terms were retained in the final model structure as they were either significant (as tested using likelihood ratio tests) or terms of interest (sample origin). X^2^ and associated *p* values are from an LRT comparing models including or excluding the effect of the tested variable, e.g. sample location (‘Bolus’). Estimates are expressed on the log scale. Intercept corresponds to edge samples originating from the first faecal bolus, for female elephants, aged 5-16 years (age category 2) and for samples collected from Kawlin camp. The model included individual elephant identification number as a random effect, as well as an observation level random effect as described above. The model was fitted to observations from 20 elephants and included a total of 120 samples. For between-bolus effect of sample location, data was not collected from any elephants aged 0-4 years and so age category 1 was not represented in the model output.

| Fixed Effect Coefficients | Estimate | Std. Error | Χ^2^ | d.f. | | | | Pr(>Chisq) |
| --- | --- | --- | --- | --- | --- | --- | --- | --- |
| (Intercept) | 2.029 | 0.341 |  |  | | | |  |
| Bolus (First) | 0.000 | 0.000 | 0.974 | 2 | | | | 0.615 |
| Bolus (Middle) | -0.045 | 0.181 |  |  | | | |  |
| Bolus (Last) | -0.174 | 0.185 |  |  | | | |  |
| Age category 2: 5-16 years | 0.000 | 0.000 | 11.919 | 2 | | | | 0.003 |
| Age category 3: 17-53 years | 0.199 | 0.392 |  |  | | | |  |
| Age category 4: >53 years | -2.443 | 0.733 |  |  | | | |  |
| Sex (Female) | 0.000 | 0.000 | 8.202 | 1 | | | | 0.004 |
| Sex (Male) | -1.136 | 0.372 |  |  | | | |  |
| Camp (Kawlin) | 0.000 | 0.000 | 6.218 | 1 | | | | 0.013 |
| Camp (Katha) | -1.067 | 0.391 |  |  | | | |  |
| Sample (Edge) | 0.000 | 0.000 | 4.516 | 1 | | | | 0.034 |
| Sample (Centre) | 0.331 | 0.150 |  |  | | | |  |
| Random Effect Coefficients | Estimate | St. Dev. | Χ^2^ | d.f. | | | Pr(>Chisq) | |
| Observation | 0.290 | 0.538 | 70.874 | | 1 | | <0.001 | |
| ID | 0.467 | 0.683 | 30.335 | | 1 | <0.001 | | |

**Table S3.** All fixed and random effects estimates and standard error for the final generalized linear mixed effects model described in **Table S2**., re-levelled to change the reference category (category 2, ‘Middle’) for Bolus. The intercept now corresponds to samples originating from the middle faecal bolus produced during a single defecation, rather than the first, with all other variables kept as previously described.

| Fixed Effect Coefficients | Estimate | | Std. Error | | Χ^2^ | d.f. | | | Pr(>Chisq) |
| --- | --- | --- | --- | --- | --- | --- | --- | --- | --- |
| (Intercept) | 1.984 | | 0.341 | |  |  | | |  |
| Bolus (Middle) | 0.000 | | 0.000 | | 0.974 | 2 | | | 0.615 |
| Bolus (First) | 0.045 | | 0.181 | |  |  | | |  |
| Bolus (Last) | -0.130 | | 0.185 | |  |  | | |  |
| Age category 2: 5-16 years | 0.000 | | 0.000 | | 11.919 | 2 | | | 0.003 |
| Age category 3: 17-53 years | 0.199 | | 0.392 | |  |  | | |  |
| Age category 4: >53 years | -2.443 | | 0.733 | |  |  | | |  |
| Sex (Female) | 0.000 | | 0.000 | | 8.202 | 1 | | | 0.004 |
| Sex (Male) | -1.136 | | 0.372 | |  |  | | |  |
| Camp (Kawlin) | 0.000 | | 0.000 | | 6.218 | 1 | | | 0.013 |
| Camp (Katha) | -1.067 | | 0.391 | |  |  | | |  |
| Sample (Edge) | 0.000 | | 0.000 | | 4.516 | 1 | | | 0.034 |
| Sample (Centre) | 0.331 | | 0.150 | |  |  | | |  |
| Random Effect Coefficients | Estimate | St. Dev. | | Χ^2^ | | | d.f. | Pr(>Chisq) | |
| Observation | 0.290 | 0.538 | | 70.874 | | | 1 | <0.001 | |
| ID | 0.467 | 0.683 | | 30.335 | | | 1 | <0.001 | |

**Table S4.** All fixed and random effects estimates and standard error for the final generalized linear mixed effects model described in **Table S2**., re-levelled to change the reference category (category 3, ‘Last’) for Bolus. The intercept now corresponds to samples originating from the last faecal bolus produced during a single defecation, with all other variables as previously described.

| Fixed Effect Coefficients | Estimate | | Std. Error | | Χ^2^ | | d.f. | Pr(>Chisq) |
| --- | --- | --- | --- | --- | --- | --- | --- | --- |
| (Intercept) | 1.855 | | 0.343 | |  | |  |  |
| Bolus (Last) | 0.000 | | 0.000 | | 0.974 | | 2 | 0.615 |
| Bolus (First) | 0.175 | | 0.185 | |  | |  |  |
| Bolus (Middle) | 0.130 | | 0.185 | |  | |  |  |
| Age category 2: 5-16 years | 0.000 | | 0.000 | | 11.919 | | 2 | 0.003 |
| Age category 3: 17-53 years | 0.199 | | 0.392 | |  | |  |  |
| Age category 4: >53 years | -2.443 | | 0.733 | |  | |  |  |
| Sex (Female) | 0.000 | | 0.000 | | 8.202 | | 1 | 0.004 |
| Sex (Male) | -1.136 | | 0.372 | |  | |  |  |
| Camp (Kawlin) | 0.000 | | 0.000 | | 6.218 | | 1 | 0.013 |
| Camp (Katha) | -1.067 | | 0.391 | |  | |  |  |
| Sample (Edge) | 0.000 | | 0.000 | | 4.516 | | 1 | 0.034 |
| Sample (Centre) | 0.331 | | 0.150 | |  | |  |  |
| Random Effect Coefficients | Estimate | | | St. Dev. | . Χ^2^ | d.f. | | Pr(>Chisq) |
| Observation | 0.290 | 0.538 | | | 70.874 | 1 | | <0.001 |
| ID | 0.467 | 0.683 | | | 30.335 | 1 | | <0.001 |

**Table S5.** Fixed and random effects estimates and standard error from the final generalized linear mixed-effects model with Poisson-lognormal error structure modelling the effect of time of sample collection (‘AM’ or ‘PM’) on helminth faecal egg count (FEC). Only significant terms or terms of interest (time of collection) were retained in the final model structure. For the starting model summary table see **Table S8.** X^2^ and associated *p* values are from an LRT comparing models including or excluding the tested variable, e.g. effect of collection time (‘Time’). Estimates are expressed on the log scale. Individual elephant identification number and an observation level effect were included as random terms. The model was fitted to observations from 47 elephants and included a total of 94 samples. Intercept corresponds to samples collected before 12pm local time (AM) from elephants located in Kawlin.

| Fixed Effect Coefficients | Estimate | | Std. Error | | Χ^2^ | | d.f. | Pr(>Chisq) | |
| --- | --- | --- | --- | --- | --- | --- | --- | --- | --- |
| (Intercept) | 3.012 | | 0.001 | |  | |  |  | |
| Time of Collection (AM) | 0.000 | | 0.000 | | 1.287 | | 1 | 0.257 | |
| Time of Collection (PM) | -0.251 | | 0.001 | |  | |  |  | |
| Camp (Kawlin) | 0.000 | | 0.000 | | 13.127 | | 1 | <0.001 | |
| Camp (Katha) | -1.786 | | 0.001 | |  | |  |  | |
| Random Effect Coefficients | Estimate | | | St. Dev. | Χ^2^ | d.f. | | | Pr(>Chisq) |
| Observation | 0.807 | 0.898 | | | 423.040 | 1 | | | <0.001 |
| ID | 0.933 | 0.966 | | | 10.163 | 1 | | | 0.001 |

**Table S6.** Fixed and random effects estimates and standard error for a final generalized linear mixed-effects model with Poisson-lognormal error structure modelling the effect of storage in fixative of faecal samples (‘Fresh’, ‘10% Formalin’ or ‘10% Formol saline’) on helminth faecal egg count (FEC). Only significant terms or terms of interest (storage method) were retained in the final model structure. For the starting model summary table see **Table S9.** Estimates are expressed on the log scale. X^2^ and associated *p* values are from an LRT comparing models including or excluding the effect of the tested variable, e.g. storage method (‘Storage Method’). The intercept corresponds to samples analysed as fresh as opposed to those stored in fixative. Individual elephant identification number and an observation level effect were included as random terms. The model was fitted to observations from 33 elephants and included a total of 132 samples. For this study, data was not collected from any elephants aged 0-4 years and so age category 1 was not represented in the model output.

| Fixed Effect Coefficients | Estimate | | | Std. Error | Χ^2^ | d.f. | Pr(>Chisq) | |
| --- | --- | --- | --- | --- | --- | --- | --- | --- |
| (Intercept) | 1.408 | | | 0.204 |  |  | |  |
| Storage Method (Fresh) | 0.000 | | | 0.000 | 55.900 | 2 | | <0.001 |
| Storage Method (10% Formalin) | -1.432 | | | 0.260 |  |  | |  |
| Storage Method (10% Formol Saline) | -1.347 | | | 0.191 |  |  | |  |
| Random Effect Coefficients | Estimate | | | St. Dev | Χ^2^ | d.f. | Pr(>Chisq) | |
| Observation | 0.290 | | 0.538 | | 36.394 | 1 | <0.001 | |
| ID | 1.032 | 1.016 | | | 45.764 | 1 | <0.001 | |

**Table S7.** All fixed and random effects estimates from a starting generalized linear mixed-effects model with Poisson-lognormal error structure, modelling the effect of faecal sample origin within a single bolus (taken from the centre or edge of the bolus) on helminth faecal egg count (FEC). All significant and non-significant terms are represented. X^2^ and associated *p* values are from an LRT comparing models with or without the effect of the tested variable, e.g. sample location (‘Sample’). Estimates are expressed on the log scale. Intercept corresponds to samples originating from the edge of the faecal bolus, for female elephants, aged 0-4 years (age category 1) and located in Katha. Individual elephant identification number was included as a random effect, as was an observation level random effect (OLRE). The model was fitted to observations from 119 elephants and included a total of 474 samples with a measure of FEC.

| Fixed Effect Coefficients | Estimate | Std. Error | | Χ^2^ | | d.f. | | Pr(>Chisq) |
| --- | --- | --- | --- | --- | --- | --- | --- | --- |
| (Intercept) | 1.478 | 1.721 | |  | |  | |  |
| Sample (Edge) | 0.000 | 0.000 | | 1.651 | | 1 | | 0.199 |
| Sample (Centre) | -0.070 | 0.054 | |  | |  | |  |
| Age category 1: <5 years | 0.000 | 0.000 | | 49.418 | | 3 | | <0.001 |
| Age category 2: 5-16 years | 1.007 | 1.690 | |  | |  | |  |
| Age category 3: 17-53 years | 0.280 | 1.708 | |  | |  | |  |
| Age category 4: >53 years | -0.546 | 1.766 | |  | |  | |  |
| Sex (Female) | 0.000 | 0.000 | | 2.410 | | 1 | | 0.121 |
| Sex (Male) | -0.494 | 0.315 | |  | |  | |  |
| Camp (Katha) | 0.000 | 0.000 | | 8.044 | | 1 | | 0.005 |
| Camp (Kawlin) | -0.882 | 0.307 | |  | |  | |  |
| Random Effect Coefficients | Estimate | | St. Dev. | Χ^2^ | d.f. | | Pr(>Chisq) | |
| Observation | 0.167 | | 0.409 | 241.370 | 1 | | <0.001 | |
| ID | 2.531 | | 1.591 | 474.520 | 1 | | <0.001 | |

**Table S8.** All fixed and random effects estimates and standard error from a starting generalized linear mixed-effects model with Poisson-lognormal error structure modelling the effect of time of sample collection (‘AM’ or ‘PM’) on helminth faecal egg count (FEC). All significant and non-significant terms are represented. X^2^ and associated *p* values are from an LRT comparing models including or excluding the effect of the tested variable, e.g. collection time (‘Time’). Estimates are expressed on the log scale. Individual elephant identification number and an observation level effect were included as random terms. The model was fitted to observations from 47 elephants and included a total of 94 samples. Intercept corresponds to samples collected before 12pm local time (AM) for female elephants located in Kawlin.

| Fixed Effect Coefficients | Estimate | Std. Error | | Χ^2^ | d.f. | | Pr(>Chisq) | |
| --- | --- | --- | --- | --- | --- | --- | --- | --- |
| (Intercept) | 2.850 | 0.706 | |  |  | |  | |
| Time of Collection (AM) | 0.000 | 0.000 | | 1.291 | 1 | | 0.256 | |
| Time of Collection (PM) | -0.251 | 0.220 | |  |  | |  | |
| Age (continuous) | 0.016 | 0.056 | | 0.078 | 1 | | 0.780 | |
| Age (quadratic) | -0.002e-^01^ | 0.009e-^01^ | | 0.058 | 1 | | 0.810 | |
| Sex (Female) | 0.000 | 0.000 | | 0.001 | 1 | | 0.971 | |
| Sex (Male) | -0.013 | 0.366 | |  |  | |  | |
| Camp (Kawlin) | 0.000 | 0.000 | | 12.486 | 1 | | <0.001 | |
| Camp (Katha) | -1.772 | 0.451 | |  |  | |  | |
| Random Effect Coefficients | Estimate | | St. Dev. | Χ^2^ | | d.f. | | Pr(>Chisq) |
| Observation | 0.807 | | 0.899 | 423.120 | | 1 | | <0.001 |
| ID | 0.938 | | 0.968 | 10.147 | | 1 | | 0.001 |

**Table S9.** All fixed and random effects estimates and standard error for a starting final generalized linear mixed-effects model with Poisson-lognormal error structure modelling the effect of storage in fixative of faecal samples (‘Fresh’, ‘10% Formalin’ or ‘10% Formol saline’) on helminth faecal egg count (FEC). All significant and non-significant terms are represented. Estimates are expressed on the log scale. X^2^ and associated *p* values are from an LRT comparing models including or excluding the effect of the tested variable, e.g. storage method (‘Storage Method’). The intercept corresponds to edge samples analysed as fresh as opposed to those stored in fixative, for female elephants, aged 5-16 years (age category 2) and located in Kawlin. Individual elephant identification number and an observation level effect were included as random terms. The model was fitted to observations from 33 elephants and included a total of 132 samples. For this study, data was not collected from any elephants aged 0-4 years and so age category 1 was not represented in the model output.

| Fixed Effect Coefficients | Estimate | Std. Error | | Χ^2^ | d.f. | | Pr(>Chisq) | |
| --- | --- | --- | --- | --- | --- | --- | --- | --- |
| (Intercept) | 2.045 | 0.468 | |  |  | |  | |
| Storage Method (Fresh) | 0.000 | 0.000 | | 56.214 | 2 | | <0.001 | |
| Storage Method (10% Formalin) | -1.480 | 0.262 | |  |  | |  | |
| Storage Method (10% Formol Saline) | -1.327 | 0.193 | |  |  | |  | |
| Age category 2: 5-16 years | 0.000 | 0.000 | | 5.678 | 2 | | 0.059 | |
| Age category 3: 17-53 years | -1.044 | 0.485 | |  |  | |  | |
| Age category 4: >53 years | -0.800 | 0.487 | |  |  | |  | |
| Sex (Female) | 0.000 | 0.000 | | 0.160 | 1 | | 0.690 | |
| Sex (Male) | 0.143 | 0.357 | |  |  | |  | |
| Camp (Kawlin) | 0.000 | 0.000 | | 1.836 | 1 | | 0.175 | |
| Camp (Katha) | -0.575 | 0.418 | |  |  | |  | |
| Sample (Edge) | 0.000 | 0.000 | | 0.574 | 1 | | 0.449 | |
| Sample (Centre) | 0.112 | 0.417 | |  |  | |  | |
| Random Effect Coefficients | Estimate | | St. Dev. | Χ^2^ | | d.f. | | Pr(>Chisq) |
| Observation | 0.294 | | 0.543 | 36.942 | | 1 | | <0.001 |
| ID | 0.801 | | 0.895 | 33.763 | | 1 | | <0.001 |
